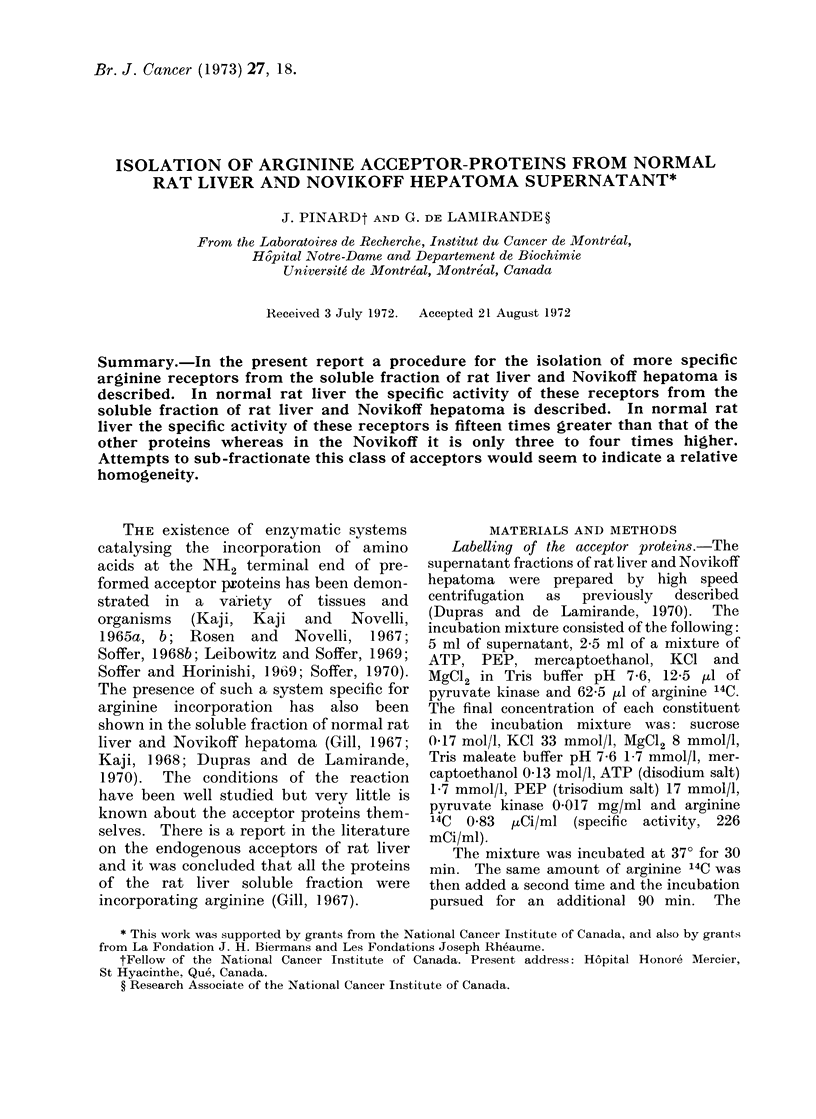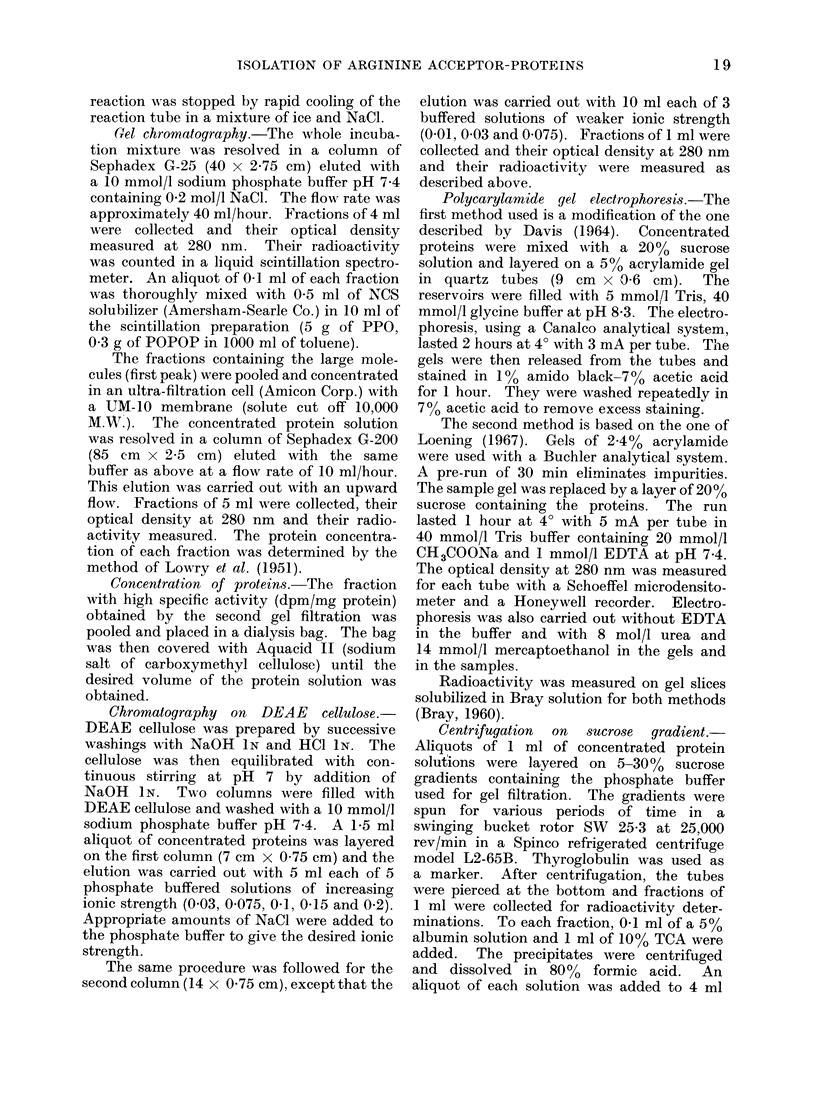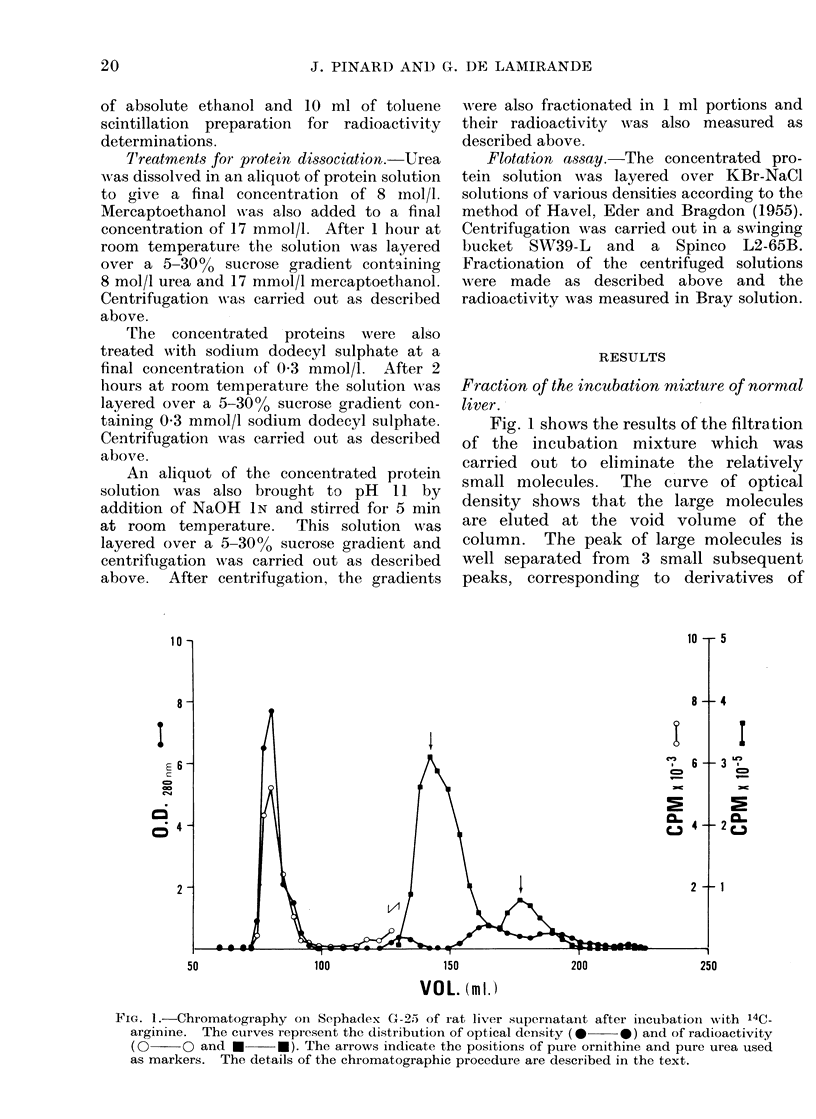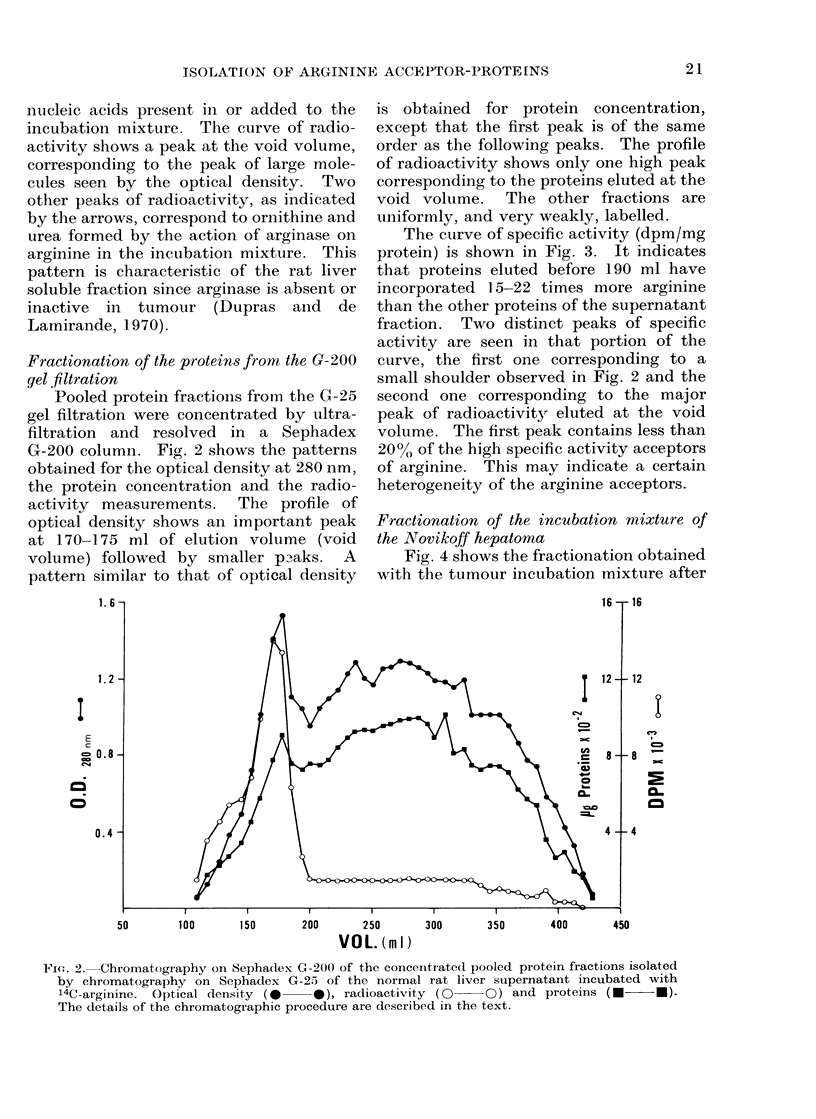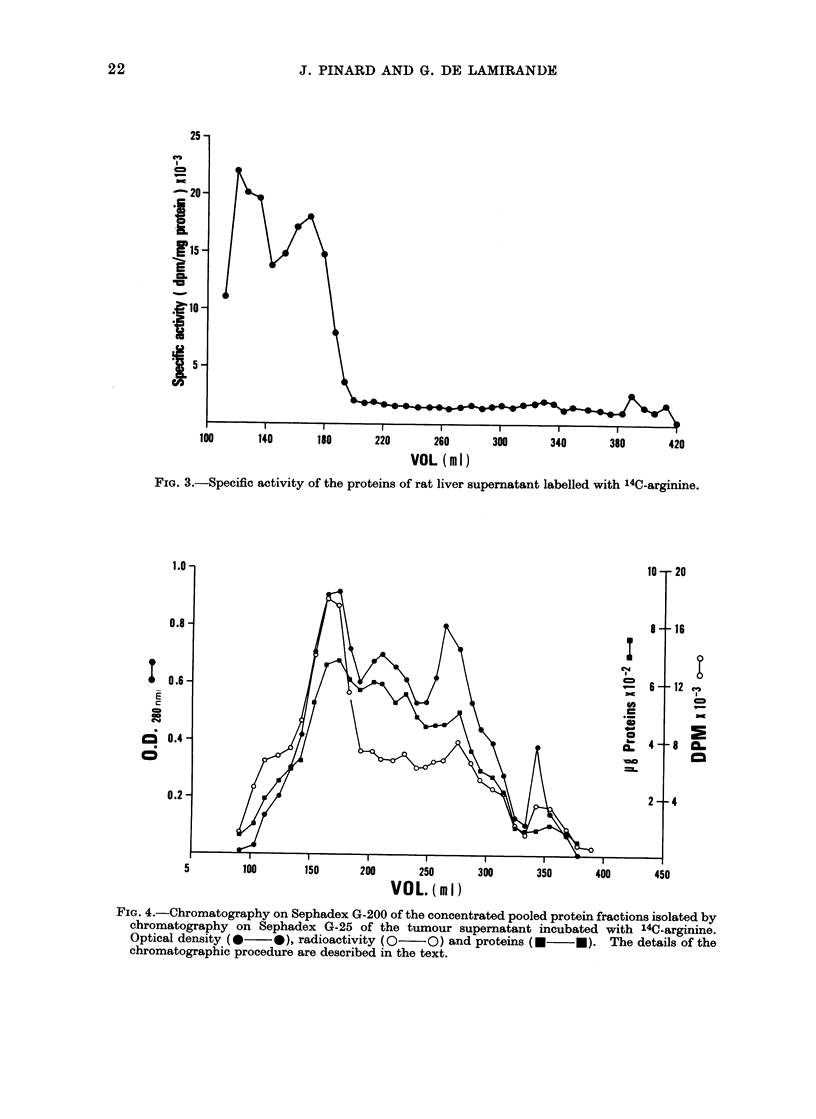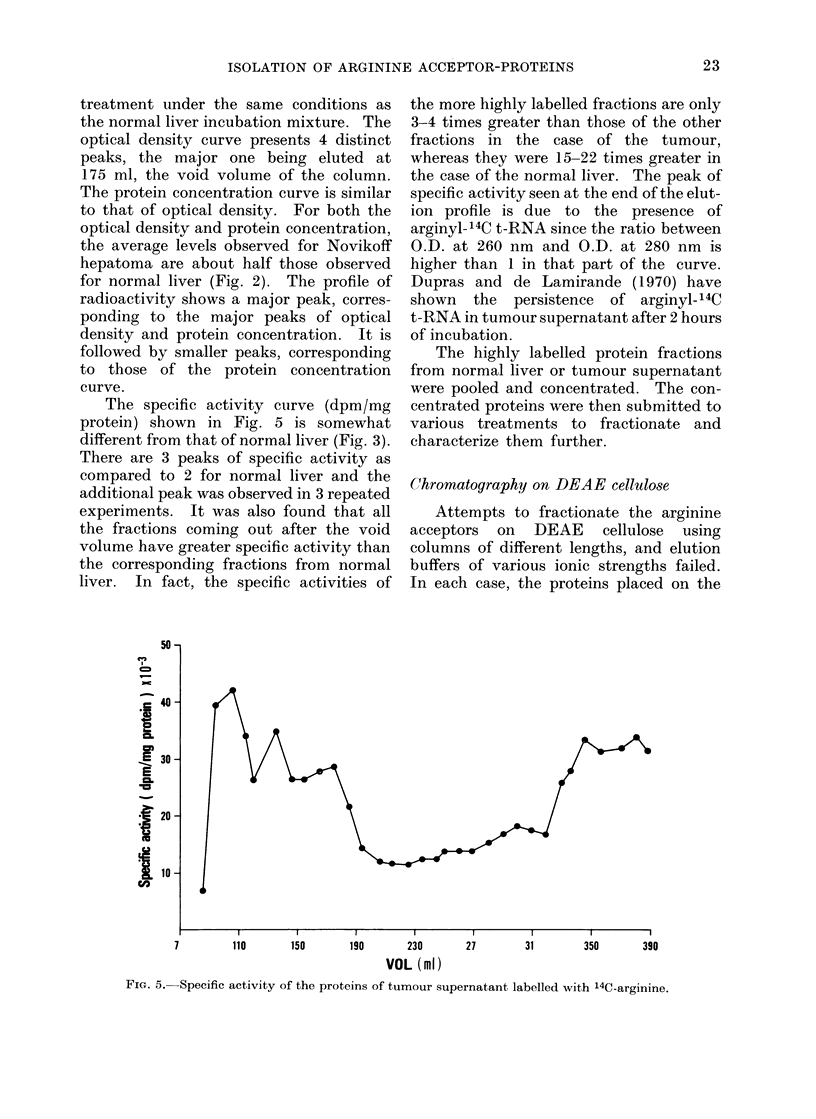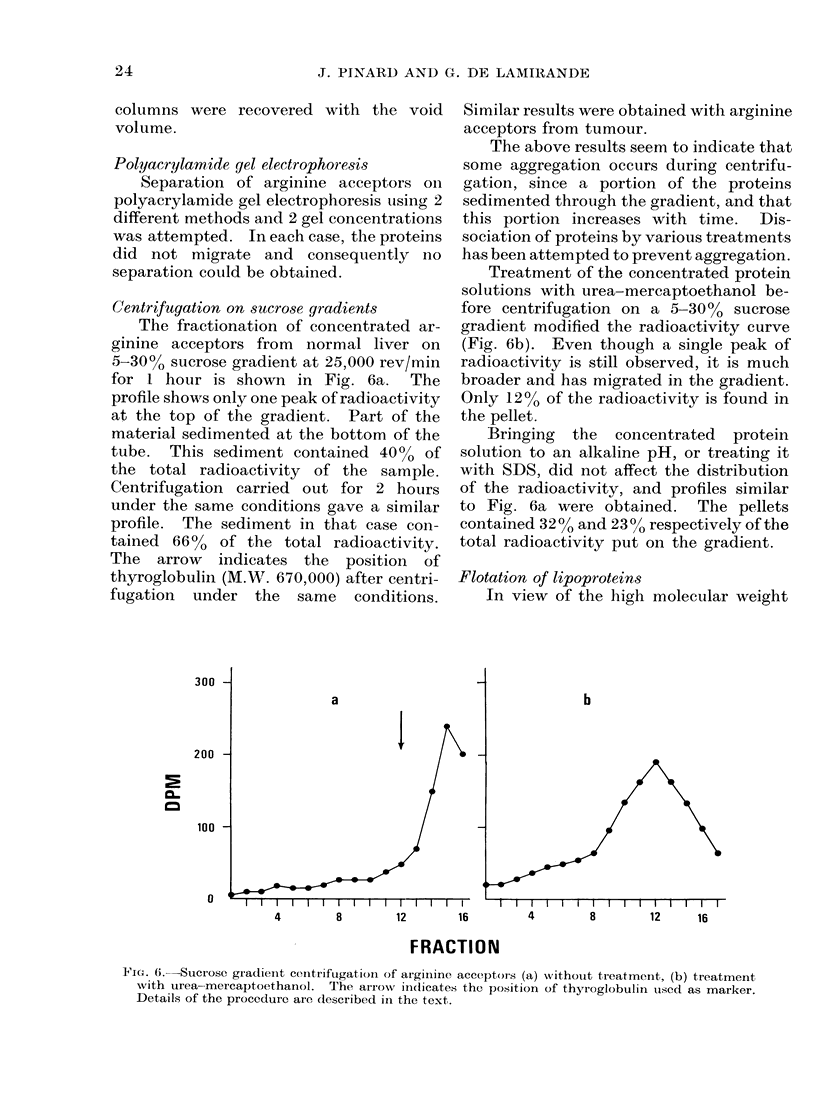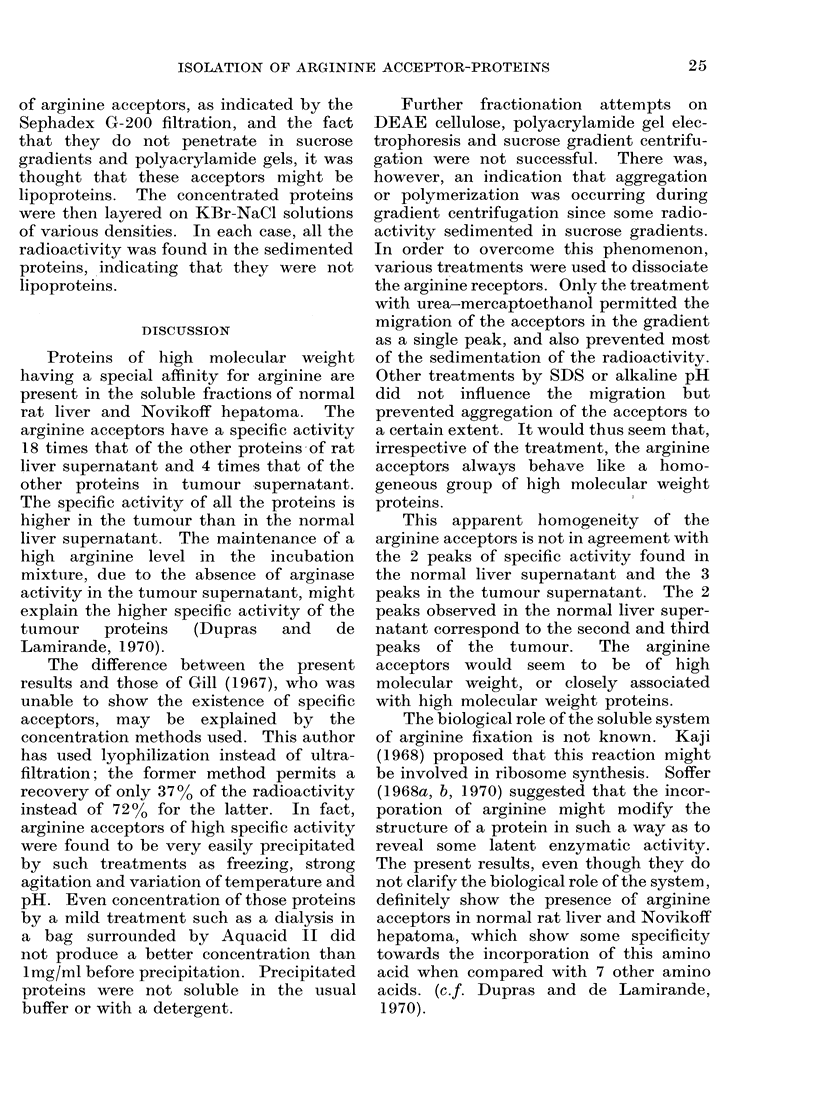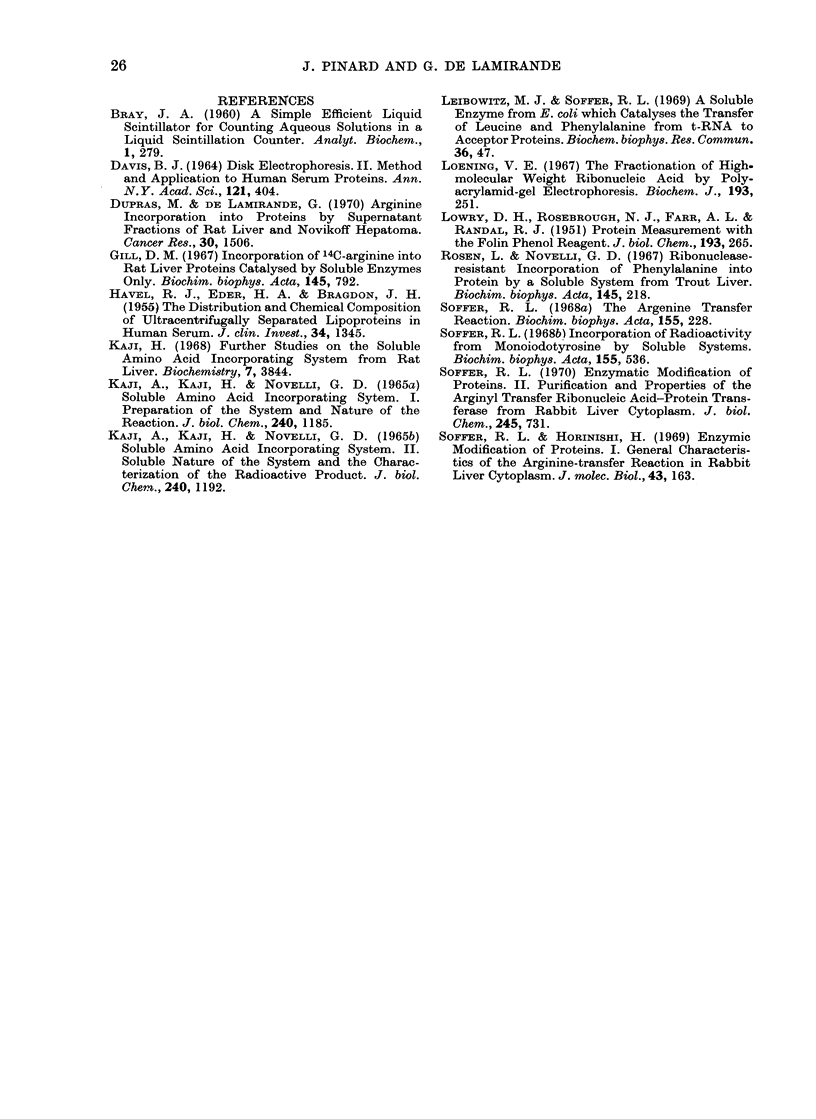# Isolation of Arginine Acceptor-Proteins from Normal Rat Liver and Novikoff Hepatoma Supernatant*

**DOI:** 10.1038/bjc.1973.3

**Published:** 1973-01

**Authors:** J. Pinard, G. de Lamirande

## Abstract

In the present report a procedure for the isolation of more specific arginine receptors from the soluble fraction of rat liver and Novikoff hepatoma is described. In normal rat liver the specific activity of these receptors from the soluble fraction of rat liver and Novikoff hepatoma is described. In normal rat liver the specific activity of these receptors is fifteen times greater than that of the other proteins whereas in the Novikoff it is only three to four times higher. Attempts to sub-fractionate this class of acceptors would seem to indicate a relative homogeneity.


*Br. J. Cancer* (1973) **27**, 18.

### ISOLATION OF ARGININE ACCEPTOR-PROTEINS FROM NORMAL RAT LIVER AND NOVIKOFF HEPATOMA SUPERNATANT*

J. PINARD† AND G. DE LAMIRANDE§

*From the Laboratoires de Recherche, Institut du Cancer de Montréal, Hôpital Notre-Dame and Departement de Biochimie Université de Montréal, Montréal, Canada*

Received 3 July 1972. Accepted 21 August 1972

**Summary.—In the present report a procedure for the isolation of more specific arginine receptors from the soluble fraction of rat liver and Novikoff hepatoma is described. In normal rat liver the specific activity of these receptors from the soluble fraction of rat liver and Novikoff hepatoma is described. In normal rat liver the specific activity of these receptors is fifteen times greater than that of the other proteins whereas in the Novikoff it is only three to four times higher. Attempts to sub-fractionate this class of acceptors would seem to indicate a relative homogeneity.**

THE existence of enzymatic systems catalysing the incorporation of amino acids at the $NH_2$ terminal end of pre-formed acceptor proteins has been demonstrated in a variety of tissues and organisms (Kaji, Kaji and Novelli, 1965*a*, *b*; Rosen and Novelli, 1967; Soffer, 1968*b*; Leibowitz and Soffer, 1969; Soffer and Horinishi, 1969; Soffer, 1970). The presence of such a system specific for arginine incorporation has also been shown in the soluble fraction of normal rat liver and Novikoff hepatoma (Gill, 1967; Kaji, 1968; Dupras and de Lamirande, 1970). The conditions of the reaction have been well studied but very little is known about the acceptor proteins themselves. There is a report in the literature on the endogenous acceptors of rat liver and it was concluded that all the proteins of the rat liver soluble fraction were incorporating arginine (Gill, 1967).

### MATERIALS AND METHODS

*Labelling of the acceptor proteins.*—The supernatant fractions of rat liver and Novikoff hepatoma were prepared by high speed centrifugation as previously described (Dupras and de Lamirande, 1970). The incubation mixture consisted of the following: 5 ml of supernatant, 2·5 ml of a mixture of ATP, PEP, mercaptoethanol, KCl and $MgCl_2$ in Tris buffer pH 7·6, 12·5 $\mu$l of pyruvate kinase and 62·5 $\mu$l of arginine $^{14}C$. The final concentration of each constituent in the incubation mixture was: sucrose 0·17 mol/l, KCl 33 mmol/l, $MgCl_2$ 8 mmol/l, Tris maleate buffer pH 7·6 1·7 mmol/l, mercaptoethanol 0·13 mol/l, ATP (disodium salt) 1·7 mmol/l, PEP (trisodium salt) 17 mmol/l, pyruvate kinase 0·017 mg/ml and arginine $^{14}C$ 0·83 $\mu$Ci/ml (specific activity, 226 mCi/ml).

The mixture was incubated at 37° for 30 min. The same amount of arginine $^{14}C$ was then added a second time and the incubation pursued for an additional 90 min. The

* This work was supported by grants from the National Cancer Institute of Canada, and also by grants from La Fondation J. H. Biermans and Les Fondations Joseph Rhéaume.

†Fellow of the National Cancer Institute of Canada. Present address: Hôpital Honoré Mercier, St Hyacinthe, Qué, Canada.

§ Research Associate of the National Cancer Institute of Canada.

reaction was stopped by rapid cooling of the reaction tube in a mixture of ice and NaCl.

*Gel chromatography.*—The whole incubation mixture was resolved in a column of Sephadex G-25 (40 × 2·75 cm) eluted with a 10 mmol/l sodium phosphate buffer pH 7·4 containing 0·2 mol/l NaCl. The flow rate was approximately 40 ml/hour. Fractions of 4 ml were collected and their optical density measured at 280 nm. Their radioactivity was counted in a liquid scintillation spectrometer. An aliquot of 0·1 ml of each fraction was thoroughly mixed with 0·5 ml of NCS solubilizer (Amersham-Searle Co.) in 10 ml of the scintillation preparation (5 g of PPO, 0·3 g of POPOP in 1000 ml of toluene).

The fractions containing the large molecules (first peak) were pooled and concentrated in an ultra-filtration cell (Amicon Corp.) with a UM-10 membrane (solute cut off 10,000 M.W.). The concentrated protein solution was resolved in a column of Sephadex G-200 (85 cm × 2·5 cm) eluted with the same buffer as above at a flow rate of 10 ml/hour. This elution was carried out with an upward flow. Fractions of 5 ml were collected, their optical density at 280 nm and their radioactivity measured. The protein concentration of each fraction was determined by the method of Lowry *et al.* (1951).

*Concentration of proteins.*—The fraction with high specific activity (dpm/mg protein) obtained by the second gel filtration was pooled and placed in a dialysis bag. The bag was then covered with Aquacid II (sodium salt of carboxymethyl cellulose) until the desired volume of the protein solution was obtained.

*Chromatography on DEAE cellulose.*—DEAE cellulose was prepared by successive washings with NaOH 1N and HCl 1N. The cellulose was then equilibrated with continuous stirring at pH 7 by addition of NaOH 1N. Two columns were filled with DEAE cellulose and washed with a 10 mmol/l sodium phosphate buffer pH 7·4. A 1·5 ml aliquot of concentrated proteins was layered on the first column (7 cm × 0·75 cm) and the elution was carried out with 5 ml each of 5 phosphate buffered solutions of increasing ionic strength (0·03, 0·075, 0·1, 0·15 and 0·2). Appropriate amounts of NaCl were added to the phosphate buffer to give the desired ionic strength.

The same procedure was followed for the second column (14 × 0·75 cm), except that the elution was carried out with 10 ml each of 3 buffered solutions of weaker ionic strength (0·01, 0·03 and 0·075). Fractions of 1 ml were collected and their optical density at 280 nm and their radioactivity were measured as described above.

*Polycarylamide gel electrophoresis.*—The first method used is a modification of the one described by Davis (1964). Concentrated proteins were mixed with a 20% sucrose solution and layered on a 5% acrylamide gel in quartz tubes (9 cm × 0·6 cm). The reservoirs were filled with 5 mmol/l Tris, 40 mmol/l glycine buffer at pH 8·3. The electrophoresis, using a Canalco analytical system, lasted 2 hours at 4° with 3 mA per tube. The gels were then released from the tubes and stained in 1% amido black–7% acetic acid for 1 hour. They were washed repeatedly in 7% acetic acid to remove excess staining.

The second method is based on the one of Loening (1967). Gels of 2·4% acrylamide were used with a Buchler analytical system. A pre-run of 30 min eliminates impurities. The sample gel was replaced by a layer of 20% sucrose containing the proteins. The run lasted 1 hour at 4° with 5 mA per tube in 40 mmol/l Tris buffer containing 20 mmol/l $CH_3COONa$ and 1 mmol/l EDTA at pH 7·4. The optical density at 280 nm was measured for each tube with a Schoeffel microdensitometer and a Honeywell recorder. Electrophoresis was also carried out without EDTA in the buffer and with 8 mol/l urea and 14 mmol/l mercaptoethanol in the gels and in the samples.

Radioactivity was measured on gel slices solubilized in Bray solution for both methods (Bray, 1960).

*Centrifugation on sucrose gradient.*—Aliquots of 1 ml of concentrated protein solutions were layered on 5–30% sucrose gradients containing the phosphate buffer used for gel filtration. The gradients were spun for various periods of time in a swinging bucket rotor SW 25·3 at 25,000 rev/min in a Spinco refrigerated centrifuge model L2-65B. Thyroglobulin was used as a marker. After centrifugation, the tubes were pierced at the bottom and fractions of 1 ml were collected for radioactivity determinations. To each fraction, 0·1 ml of a 5% albumin solution and 1 ml of 10% TCA were added. The precipitates were centrifuged and dissolved in 80% formic acid. An aliquot of each solution was added to 4 ml of absolute ethanol and 10 ml of toluene scintillation preparation for radioactivity determinations.

*Treatments for protein dissociation.*—Urea was dissolved in an aliquot of protein solution to give a final concentration of 8 mol/l. Mercaptoethanol was also added to a final concentration of 17 mmol/l. After 1 hour at room temperature the solution was layered over a 5–30% sucrose gradient containing 8 mol/l urea and 17 mmol/l mercaptoethanol. Centrifugation was carried out as described above.

The concentrated proteins were also treated with sodium dodecyl sulphate at a final concentration of 0·3 mmol/l. After 2 hours at room temperature the solution was layered over a 5–30% sucrose gradient containing 0·3 mmol/l sodium dodecyl sulphate. Centrifugation was carried out as described above.

An aliquot of the concentrated protein solution was also brought to pH 11 by addition of NaOH 1N and stirred for 5 min at room temperature. This solution was layered over a 5–30% sucrose gradient and centrifugation was carried out as described above. After centrifugation, the gradients were also fractionated in 1 ml portions and their radioactivity was also measured as described above.

*Flotation assay.*—The concentrated protein solution was layered over KBr-NaCl solutions of various densities according to the method of Havel, Eder and Bragdon (1955). Centrifugation was carried out in a swinging bucket SW39-L and a Spinco L2-65B. Fractionation of the centrifuged solutions were made as described above and the radioactivity was measured in Bray solution.

### RESULTS

*Fraction of the incubation mixture of normal liver.*

Fig. 1 shows the results of the filtration of the incubation mixture which was carried out to eliminate the relatively small molecules. The curve of optical density shows that the large molecules are eluted at the void volume of the column. The peak of large molecules is well separated from 3 small subsequent peaks, corresponding to derivatives of

Fig. 1.—Chromatography on Sephadex G-25 of rat liver supernatant after incubation with $^{14}C$-arginine. The curves represent the distribution of optical density (●——●) and of radioactivity (○——○ and ■——■). The arrows indicate the positions of pure ornithine and pure urea used as markers. The details of the chromatographic procedure are described in the text.

nucleic acids present in or added to the incubation mixture. The curve of radioactivity shows a peak at the void volume, corresponding to the peak of large molecules seen by the optical density. Two other peaks of radioactivity, as indicated by the arrows, correspond to ornithine and urea formed by the action of arginase on arginine in the incubation mixture. This pattern is characteristic of the rat liver soluble fraction since arginase is absent or inactive in tumour (Dupras and de Lamirande, 1970).

*Fractionation of the proteins from the G-200 gel filtration*

Pooled protein fractions from the G-25 gel filtration were concentrated by ultrafiltration and resolved in a Sephadex G-200 column. Fig. 2 shows the patterns obtained for the optical density at 280 nm, the protein concentration and the radioactivity measurements. The profile of optical density shows an important peak at 170–175 ml of elution volume (void volume) followed by smaller p
eaks. A pattern similar to that of optical density is obtained for protein concentration, except that the first peak is of the same order as the following peaks. The profile of radioactivity shows only one high peak corresponding to the proteins eluted at the void volume. The other fractions are uniformly, and very weakly, labelled.

The curve of specific activity (dpm/mg protein) is shown in Fig. 3. It indicates that proteins eluted before 190 ml have incorporated 15–22 times more arginine than the other proteins of the supernatant fraction. Two distinct peaks of specific activity are seen in that portion of the curve, the first one corresponding to a small shoulder observed in Fig. 2 and the second one corresponding to the major peak of radioactivity eluted at the void volume. The first peak contains less than 20% of the high specific activity acceptors of arginine. This may indicate a certain heterogeneity of the arginine acceptors.

*Fractionation of the incubation mixture of the Novikoff hepatoma*

Fig. 4 shows the fractionation obtained with the tumour incubation mixture after

FIG. 2.—Chromatography on Sephadex G-200 of the concentrated pooled protein fractions isolated by chromatography on Sephadex G-25 of the normal rat liver supernatant incubated with $^{14}C$-arginine. Optical density (●——●), radioactivity (○——○) and proteins (■——■). The details of the chromatographic procedure are described in the text.

FIG. 3.—Specific activity of the proteins of rat liver supernatant labelled with $^{14}C$-arginine.

FIG. 4.—Chromatography on Sephadex G-200 of the concentrated pooled protein fractions isolated by chromatography on Sephadex G-25 of the tumour supernatant incubated with $^{14}C$-arginine. Optical density (●——●), radioactivity (○——○) and proteins (■——■). The details of the chromatographic procedure are described in the text.

treatment under the same conditions as the normal liver incubation mixture. The optical density curve presents 4 distinct peaks, the major one being eluted at 175 ml, the void volume of the column. The protein concentration curve is similar to that of optical density. For both the optical density and protein concentration, the average levels observed for Novikoff hepatoma are about half those observed for normal liver (Fig. 2). The profile of radioactivity shows a major peak, corresponding to the major peaks of optical density and protein concentration. It is followed by smaller peaks, corresponding to those of the protein concentration curve.

The specific activity curve (dpm/mg protein) shown in Fig. 5 is somewhat different from that of normal liver (Fig. 3). There are 3 peaks of specific activity as compared to 2 for normal liver and the additional peak was observed in 3 repeated experiments. It was also found that all the fractions coming out after the void volume have greater specific activity than the corresponding fractions from normal liver. In fact, the specific activities of the more highly labelled fractions are only 3–4 times greater than those of the other fractions in the case of the tumour, whereas they were 15–22 times greater in the case of the normal liver. The peak of specific activity seen at the end of the elution profile is due to the presence of arginyl-$^{14}C$ t-RNA since the ratio between O.D. at 260 nm and O.D. at 280 nm is higher than 1 in that part of the curve. Dupras and de Lamirande (1970) have shown the persistence of arginyl-$^{14}C$ t-RNA in tumour supernatant after 2 hours of incubation.

The highly labelled protein fractions from normal liver or tumour supernatant were pooled and concentrated. The concentrated proteins were then submitted to various treatments to fractionate and characterize them further.

### *Chromatography on DEAE cellulose*

Attempts to fractionate the arginine acceptors on DEAE cellulose using columns of different lengths, and elution buffers of various ionic strengths failed. In each case, the proteins placed on the

FIG. 5.—Specific activity of the proteins of tumour supernatant labelled with $^{14}C$-arginine.

columns were recovered with the void volume.

*Polyacrylamide gel electrophoresis*

Separation of arginine acceptors on polyacrylamide gel electrophoresis using 2 different methods and 2 gel concentrations was attempted. In each case, the proteins did not migrate and consequently no separation could be obtained.

*Centrifugation on sucrose gradients*

The fractionation of concentrated arginine acceptors from normal liver on 5–30% sucrose gradient at 25,000 rev/min for 1 hour is shown in Fig. 6a. The profile shows only one peak of radioactivity at the top of the gradient. Part of the material sedimented at the bottom of the tube. This sediment contained 40% of the total radioactivity of the sample. Centrifugation carried out for 2 hours under the same conditions gave a similar profile. The sediment in that case contained 66% of the total radioactivity. The arrow indicates the position of thyroglobulin (M.W. 670,000) after centrifugation under the same conditions. Similar results were obtained with arginine acceptors from tumour.

The above results seem to indicate that some aggregation occurs during centrifugation, since a portion of the proteins sedimented through the gradient, and that this portion increases with time. Dissociation of proteins by various treatments has been attempted to prevent aggregation.

Treatment of the concentrated protein solutions with urea–mercaptoethanol before centrifugation on a 5–30% sucrose gradient modified the radioactivity curve (Fig. 6b). Even though a single peak of radioactivity is still observed, it is much broader and has migrated in the gradient. Only 12% of the radioactivity is found in the pellet.

Bringing the concentrated protein solution to an alkaline pH, or treating it with SDS, did not affect the distribution of the radioactivity, and profiles similar to Fig. 6a were obtained. The pellets contained 32% and 23% respectively of the total radioactivity put on the gradient.

*Flotation of lipoproteins*

In view of the high molecular weight

FIG. 6.—Sucrose gradient centrifugation of arginine acceptors (a) without treatment, (b) treatment with urea–mercaptoethanol. The arrow indicates the position of thyroglobulin used as marker. Details of the procedure are described in the text.

of arginine acceptors, as indicated by the Sephadex G-200 filtration, and the fact that they do not penetrate in sucrose gradients and polyacrylamide gels, it was thought that these acceptors might be lipoproteins. The concentrated proteins were then layered on KBr-NaCl solutions of various densities. In each case, all the radioactivity was found in the sedimented proteins, indicating that they were not lipoproteins.

### DISCUSSION

Proteins of high molecular weight having a special affinity for arginine are present in the soluble fractions of normal rat liver and Novikoff hepatoma. The arginine acceptors have a specific activity 18 times that of the other proteins of rat liver supernatant and 4 times that of the other proteins in tumour supernatant. The specific activity of all the proteins is higher in the tumour than in the normal liver supernatant. The maintenance of a high arginine level in the incubation mixture, due to the absence of arginase activity in the tumour supernatant, might explain the higher specific activity of the tumour proteins (Dupras and de Lamirande, 1970).

The difference between the present results and those of Gill (1967), who was unable to show the existence of specific acceptors, may be explained by the concentration methods used. This author has used lyophilization instead of ultrafiltration; the former method permits a recovery of only 37% of the radioactivity instead of 72% for the latter. In fact, arginine acceptors of high specific activity were found to be very easily precipitated by such treatments as freezing, strong agitation and variation of temperature and pH. Even concentration of those proteins by a mild treatment such as a dialysis in a bag surrounded by Aquacid II did not produce a better concentration than 1mg/ml before precipitation. Precipitated proteins were not soluble in the usual buffer or with a detergent.

Further fractionation attempts on DEAE cellulose, polyacrylamide gel electrophoresis and sucrose gradient centrifugation were not successful. There was, however, an indication that aggregation or polymerization was occurring during gradient centrifugation since some radioactivity sedimented in sucrose gradients. In order to overcome this phenomenon, various treatments were used to dissociate the arginine receptors. Only the treatment with urea–mercaptoethanol permitted the migration of the acceptors in the gradient as a single peak, and also prevented most of the sedimentation of the radioactivity. Other treatments by SDS or alkaline pH did not influence the migration but prevented aggregation of the acceptors to a certain extent. It would thus seem that, irrespective of the treatment, the arginine acceptors always behave like a homogeneous group of high molecular weight proteins.

This apparent homogeneity of the arginine acceptors is not in agreement with the 2 peaks of specific activity found in the normal liver supernatant and the 3 peaks in the tumour supernatant. The 2 peaks observed in the normal liver supernatant correspond to the second and third peaks of the tumour. The arginine acceptors would seem to be of high molecular weight, or closely associated with high molecular weight proteins.

The biological role of the soluble system of arginine fixation is not known. Kaji (1968) proposed that this reaction might be involved in ribosome synthesis. Soffer (1968*a*, *b*, 1970) suggested that the incorporation of arginine might modify the structure of a protein in such a way as to reveal some latent enzymatic activity. The present results, even though they do not clarify the biological role of the system, definitely show the presence of arginine acceptors in normal rat liver and Novikoff hepatoma, which show some specificity towards the incorporation of this amino acid when compared with 7 other amino acids. (*c.f.* Dupras and de Lamirande, 1970).

REFERENCES

Bray, J. A. (1960) A Simple Efficient Liquid Scintillator for Counting Aqueous Solutions in a Liquid Scintillation Counter. *Analyt. Biochem.*, **1**, 279.

Davis, B. J. (1964) Disk Electrophoresis. II. Method and Application to Human Serum Proteins. *Ann. N.Y. Acad. Sci.*, **121**, 404.

Dupras, M. & de Lamirande, G. (1970) Arginine Incorporation into Proteins by Supernatant Fractions of Rat Liver and Novikoff Hepatoma. *Cancer Res.*, **30**, 1506.

Gill, D. M. (1967) Incorporation of $^{14}$C-arginine into Rat Liver Proteins Catalysed by Soluble Enzymes Only. *Biochim. biophys. Acta*, **145**, 792.

Havel, R. J., Eder, H. A. & Bragdon, J. H. (1955) The Distribution and Chemical Composition of Ultracentrifugally Separated Lipoproteins in Human Serum. *J. clin. Invest.*, **34**, 1345.

Kaji, H. (1968) Further Studies on the Soluble Amino Acid Incorporating System from Rat Liver. *Biochemistry*, **7**, 3844.

Kaji, A., Kaji, H. & Novelli, G. D. (1965*a*) Soluble Amino Acid Incorporating Sytem. I. Preparation of the System and Nature of the Reaction. *J. biol. Chem.*, **240**, 1185.

Kaji, A., Kaji, H. & Novelli, G. D. (1965*b*) Soluble Amino Acid Incorporating System. II. Soluble Nature of the System and the Characterization of the Radioactive Product. *J. biol. Chem.*, **240**, 1192.

Leibowitz, M. J. & Soffer, R. L. (1969) A Soluble Enzyme from *E. coli* which Catalyses the Transfer of Leucine and Phenylalanine from t-RNA to Acceptor Proteins. *Biochem. biophys. Res. Commun.* **36**, 47.

Loening, V. E. (1967) The Fractionation of High-molecular Weight Ribonucleic Acid by Poly-acrylamid-gel Electrophoresis. *Biochem. J.*, **193**, 251.

Lowry, D. H., Rosebrough, N. J., Farr, A. L. & Randal, R. J. (1951) Protein Measurement with the Folin Phenol Reagent. *J. biol. Chem.*, **193**, 265.

Rosen, L. & Novelli, G. D. (1967) Ribonuclease-resistant Incorporation of Phenylalanine into Protein by a Soluble System from Trout Liver. *Biochim. biophys. Acta*, **145**, 218.

Soffer, R. L. (1968*a*) The Argenine Transfer Reaction. *Biochim. biophys. Acta*, **155**, 228.

Soffer, R. L. (1968*b*) Incorporation of Radioactivity from Monoiodotyrosine by Soluble Systems. *Biochim. biophys. Acta*, **155**, 536.

Soffer, R. L. (1970) Enzymatic Modification of Proteins. II. Purification and Properties of the Arginyl Transfer Ribonucleic Acid–Protein Transferase from Rabbit Liver Cytoplasm. *J. biol. Chem.*, **245**, 731.

Soffer, R. L. & Horinishi, H. (1969) Enzymic Modification of Proteins. I. General Characteristics of the Arginine-transfer Reaction in Rabbit Liver Cytoplasm. *J. molec. Biol.*, **43**, 163.